# Impact of cannabis use on white matter hyperintensities in adults with and without chronic HIV disease

**DOI:** 10.1007/s13365-026-01338-2

**Published:** 2026-08-27

**Authors:** Sreelakshmi Shaji, Sheri L. Towe, Paul J. Laurienti, Robert G. Lyday, Jonathan H. Burdette, Christina S. Meade

**Affiliations:** 1 Department of Translational Neuroscience, Wake Forest University School of Medicine, Winston-Salem, NC USA; 2 Department of Radiology, Wake Forest University School of Medicine, Winston-Salem, NC USA

**Keywords:** Cannabis, HIV disease, White matter hyperintensities, Cognitive function

## Abstract

White matter hyperintensities (WMH) are prevalent among people living with HIV (PLWH), even with sustained viral suppression on antiretroviral therapy. This study investigated whether cannabis use contributes to additional WMH load among PLWH and its association to neurocognitive function. A total of 296 adult participants, stratified by HIV and cannabis status (74 control, 73 cannabis-only, 76 HIV-only and 73 HIV+cannabis) were enrolled. High resolution anatomical MRI and fluid-attenuated inversion recovery (FLAIR) images were collected, along with a battery of neuropsychological tests. Periventricular (pvWMH) and deep (dWMH) WMH loads were quantified using an automated pipeline. Statistical tests were employed to examine the main and interaction effects of HIV and cannabis use on WMH, and to explore the association of WMH loads with neuropsychological performance. The cohort had a mean age of 39.84 years, and all PLWH had sustained viral suppression. pvWMH loads were higher in the cannabis-only and HIV-only groups compared to controls. A significant interaction effect of HIV by cannabis was found for pvWMH load, such that participants with co-occurring HIV and cannabis use had similar pvWMH load compared to controls. In addition, increased pvWMH load significantly correlated with lower cognitive performance. Our findings suggest that cannabis use does not contribute additively to WMH burden in PLWH and may potentially be associated with a reduced WMH burden. However, future work is warranted to substantiate this finding, particularly with respect to type and levels of circulating cannabinoid metabolites.

**Supplementary Information:**

The online version contains supplementary material available at https://doi.org/10.1007/s13365-026-01338-2.

## Introduction

Cannabis use is common among people living with HIV (PLWH), with approximately 77% reporting lifetime use. Moreover, a substantial proportion ranging from 18 to 34% report cannabis use during the past year (Montgomery et al. 2019). Although it is demonstrated to alleviate HIV associated symptoms, such as nausea, pain, and fatigue, the definitive therapeutic benefits of cannabis for HIV disease remain elusive (Chen et al. 2026). Importantly, emerging evidence suggests that cannabinoids may modulate immune and inflammatory processes, raising the possibility that cannabis use could influence the persistent neuroinflammation that characterizes HIV infection and, consequently, affect brain health outcomes in PLWH (Ellison et al. 2026; Langat et al. 2025). However, despite these potential neurobiological effects, studies examining the relationship between cannabis use and neurocognitive functioning in PLWH have yielded inconsistent results (Ayoub et al. 2024; Lorkiewicz et al. 2018; Okafor et al. 2019; Skalski et al. 2016; Thames et al. 2017; Watson et al. 2021). While some studies indicate an absence of cognitive deficits in persons who use cannabis regularly regardless of HIV status (Lorkiewicz et al. 2018; Thames et al. 2017), others have observed substantial memory impairment related to cannabis use in PLWH (Okafor et al. 2019; Watson et al. 2023). Recent studies also suggest that cannabis possesses neuroprotective properties, potentially improving cognition in various cohorts (Ayoub et al. 2024). Altogether, these findings suggest that the interplay between cannabis use and HIV disease on brain structure and cognitive function is complex.

Accumulating evidence suggests that white matter hyperintensities (WMHs) in the brain have detrimental effects on higher-order cognition, including memory and executive function (d’Arbeloff et al. 2019; Rizvi et al. 2018; Wang et al. 2022). They are histopathologically linked to cerebral small vessel disease, chronic hypoperfusion of white matter, and disruption of the blood-brain barrier (Gupta et al. 2018; Holroyd et al. 2025). Studies suggest that WMH disrupts the brain networks subserving cognitive processes and thereby is among the strongest radiological predictors of cognitive decline. These white matter abnormalities can be identified on fluid attenuated inversion recovery (FLAIR) magnetic resonance (MR) images. Depending on the anatomical distribution, WMHs are classified as periventricular (pvWMHs) when they border the lateral ventricles, and as deep (dWMHs) when they are located away from the ventricles and in subcortical regions. The primary risk factors for WMH are aging and cardiovascular diseases (CVD), such as hypertension, diabetes, and obesity (Gupta et al. 2018; Mina et al. 2021; Pfefferbaum et al. 2024).

Increases in WMH volume are consistently reported in chronic HIV disease (Chien et al. 2024; d’Arbeloff et al. 2019; Holroyd et al. 2025; Mina et al. 2021; Pfefferbaum et al. 2024). Elevated burden and accelerated progression of WMHs has been reported even among PLWH who are virally suppressed on antiretroviral therapy (ART) (Holroyd et al. 2025). However, several studies have reported no significant correlations between WMH volume and HIV disease characteristics, such as CD4^+^ and CD8^+^ T-cell count (Holroyd et al. 2025; Trentalange et al. 2020; Watson et al. 2017). As in the general population, WMH lesions among PLWH are associated with CVD, which occur at higher rates (Chien et al. 2024; McIntosh et al. 2021; Mina et al. 2021), and are predictive of neurocognitive impairment (Chien et al. 2024; Watson et al. 2017).

Independent of HIV, a few studies have revealed the presence of pvWMHs in people who use cannabis (Ishrat et al. 2024; Vered et al. 2024; Volpon et al. 2017; Wang et al. 2020). Despite the high prevalence of cannabis use in PLWH, research examining their interactive effect on brain white matter abnormalities remains scant. One study that utilized diffusion weighted imaging reported that chronic cannabis use did not exacerbate the negative impact of HIV on the white matter microstructure, with the exception of globus pallidus (Wang et al. 2020). However, the influence of cannabis use on brain WMH in PLWH is unknown.

Here, we address this gap in the literature by investigating the independent and synergistic effects of cannabis use and HIV disease on brain WMHs. Given that aging and CVD are robust predictors of WMHs, these factors were controlled in all models. Quantitative assessment of WMH volume and distribution may provide insight into the underlying mechanisms of these lesions in clinically relevant populations (Wang et al. 2022). We extend prior work by examining regional variation in pvWMH and dWMH volumes in a cross-sectional cohort of 296 adults, with and without HIV as well as with and without cannabis use. Notably, the cohort consisted of relatively young, middle-aged adults (mean age in the late 30s), an age range in which WMH burden is typically low.We also examined the association of these WMHs with domain-specific cognitive functions. We hypothesized that HIV disease and cannabis use would be independently associated with higher pvWMH burden and lower neurocognitive functioning, and that PLWH who use cannabis would have the greatest deficits relative to controls. We further anticipated that WMHs would be linked to poorer neurocognitive performance.

## Materials and methods

### Study participants and procedures

Adults aged 25–59 years were recruited from the Raleigh-Durham area of North Carolina via advertisements and flyers in community-based organizations, websites, and medical clinics. Participants were stratified by HIV status and cannabis use status, resulting in four groups: participants without HIV who do not use cannabis (control), participants without HIV who use cannabis (CU-only), PLWH who do not use cannabis (HIV-only), and PLWH who use cannabis (HIV + CU). PLWH were required to be on a current ARV regimen, to have current plasma HIV RNA < 200 copies/mL, and to have received combination antiretroviral therapy as first-line of treatment after HIV diagnosis. Participants in the CU-only and HIV + CU groups had to meet the following criteria: (1) ≥ 4 days of cannabis use in the past 30 days or a THC-positive urine drug screen, and (2) ≥ 1 year of lifetime regular cannabis use. Participants in the control and HIV-only groups were required to have 0 days cannabis use in the past 30 days, a THC-negative urine drug screen, and no regular cannabis use in the past 10 years. For all groups, alcohol and nicotine use were permitted due to their high prevalence among PLWH and people who use cannabis. For illicit drugs other than cannabis, participants were excluded for: a positive urine drug screen, > 2 days of use in the past 30 days, any regular use within the prior 2 years, ≥ 5 cumulative years of lifetime regular use, and a current (past 12 months) substance use disorder. Additional exclusion criteria were: non-fluency in English, illiteracy, severe head trauma with loss of consciousness > 30 min and persistent functional decline, neurological disorders or serious neurological events without return to normal cognition, severe mental illness (defined as acute manic or psychotic symptoms), MRI contraindications, and pregnancy.

After a brief prescreening interview to confirm preliminary eligibility, participants provided written informed consent and completed an in-person eligibility screening. The screening included questionnaires, clinical interviews, urine drug and pregnancy testing, and verification of HIV status, as well as an MRI simulation to acclimate them to the scanning procedures and assess for possible contraindications. For PLWH, HIV diagnosis was verified by medical record review. Plasma HIV RNA was assessed at the baseline study visit, and medical records were reviewed to confirm sustained viral suppression (< 200 copies/mL) during the preceding six months. Nadir CD4 cell count was defined as the lowest documented CD4 count and was obtained through review of available medical records from the time of HIV diagnosis through study enrollment. For all other participants, the screening included a rapid HIV-1/2 antibody test on a droplet of whole blood collected via finger stick. All participants screened had a non-reactive test result. Eligible participants then completed additional assessments, including an MRI brain scan and neuropsychological testing. Procedures were approved by the Institutional Review Board at Duke University Health System.

### Assessment measures

*Substance use.* The Addiction Severity Index-Lite (ASI-Lite) assessed substance use and related areas of function (McLellan et al. 1992), and substance use disorder criteria were evaluated using Module E of the Structured Clinical Interview for DSM-5 (SCID-5) (First et al. 2015). In addition, participants reported the number of days they consumed alcohol and other substances, including cannabis, during the past 30 days. The Lifetime Drug Use Questionnaire (Czermak et al. 2005) assessed the duration, frequency, and quantity of lifetime cannabis use, including the age of use initiation. The urine drug toxicology test assessed the presence of 11 different substances (cannabis, cocaine, opiates, methamphetamine, amphetamine, benzodiazepines, barbiturates, methadone, buprenorphine, ecstasy, oxycodone).

*Clinical data.* Participants provided releases to allow the study team to obtain copies of their healthcare records. This information was used to ensure the absence of exclusionary medical conditions and to abstract relevant clinical data on HIV disease characteristics for PLWH and CVD risk factors for all participants. HIV disease characteristics included date of HIV diagnosis, date of antiretroviral (ARV) initiation, and nadir CD4^+^ T-cell count. For CVD risk, the following were coded as either present or absent: obesity, diabetes, hypertension, hyperlipidemia, and nicotine use. In cases where medical records were incomplete, information was supplemented with self-reported medical history. Height and weight measured at the study visit were used to compute body mass index (BMI), and then BMI was dichotomized (BMI ≥ 30) to define obesity. Participants self-reported whether they had used any nicotine in the past 30 days. A composite CVD risk score (ranging 0 to 5) was then calculated as the sum of 5 risk factors. For PLWH, blood samples were collected by peripheral venipuncture, and samples were sent to Quest Diagnostics to test current CD4^+^ T-cell count and to confirm HIV viral suppression at ≤ 200 copies.

*Other measures.* Questionnaires assessed demographic information, including age, sex, race, and years of education. Additional measures to characterize the sample and evaluate eligibility criteria included Modules A and B of SCID-5 to assess lifetime psychiatric disorders and severe mental illness (First et al. 2015) and the word reading test of the Wide Range Achievement Test 4 (Wilkinson and Robertson 2006) to assess literacy.

### Neuropsychological assessment

Standardized neuropsychological tests assessed function across four domains:

1. Executive functioning: Delis-Kaplan Executive Function System (D-KEFS) Tower Test – total achievement score (Delis et al. 2001); Wisconsin Card Sorting Test – total errors (Kongs et al. 2000); Stroop Color and Word Test Interference – difference between actual and predicted score on the Color-Word trial (Golden 1978); Trail Making Test Part B – number of seconds to completion (Heaton et al. 1991; Reitan and Wolfson 1993).
2. Working memory: Paced Auditory Serial Addition Task-50 – total number correct (Diehr et al. 2003); WAIS-IV Digit Span – total number correct (Wechsler 2008); WAIS-IV Letter-Number Sequencing – total number correct (Wechsler 2008); WMS-IV Spatial Addition– total number correct (Wechsler 2009).
3. Learning (immediate recall): Hopkins Verbal Learning Test-Revised (HVLT-R) – total number of words recalled on trials 1–3 (Brandt and Benedict 2001); Brief Visual Memory Test-Revised (BVMT-R) – total score for figures recalled on trials 1–3 (Benedict et al. 1996); WMS-IV Logical Memory – total number of story details recalled in Trial I (Wechsler 2009).
4. Memory (delayed recall): HVLT-R – total number of words recalled on trial 4 (Brandt and Benedict 2001); BVMT-R – total score for figures recalled on trial 4 (Benedict et al. 1996); WMS-IV Logical Memory – total number of story details recalled in Trial II (Wechsler 2009).

For all tests, the most-updated scoring materials available from the test publisher were used to convert raw scores to demographically-adjusted T scores. Thus, scores are referenced to population norms (mean = 50, SD = 10), with lower scores indicating poorer performance relative to expected demographic peers. Within each domain, test T scores were then averaged to generate a mean domain T score, and then domain T scores were averaged to generate a global T score.

### MRI data acquisition

All participants underwent MRI scans using a 3.0T GE SIGNAÔ Premier whole-body scanner with a 48-channel head coil at Duke University Hospital in North Carolina, USA. High-resolution T1-weighted (T1w) images were obtained using a BRAVO sequence with a SENSE factor of 2 (repetition time [TR] = 2234.94ms, echo time [TE] = 3.076ms, inversion time [TI] = 900ms, voxel size = 0.9375 mm x 0.9375 mm x 1 mm, field of view [FOV] = 240mm^2^, 8° flip angle, 154 interleaved slices). Fast-spin echo T2-weighted FLAIR images were acquired in oblique axial orientation of the full brain. The scan parameters include a voxel size of 0.5 mm × 0.5 mm × 5 mm, TR of 10,000 msec, TE of 120 msec, TI of 2337 msec, FOV of 25.6, 111° flip angle, and 27 slices. The RX27.0 software of the scanner was upgraded to RX28.0 midway through data collection, and so software version was included as a binary covariate in all analyses.

### MRI processing and WMH quantification

**Fig. 1:**
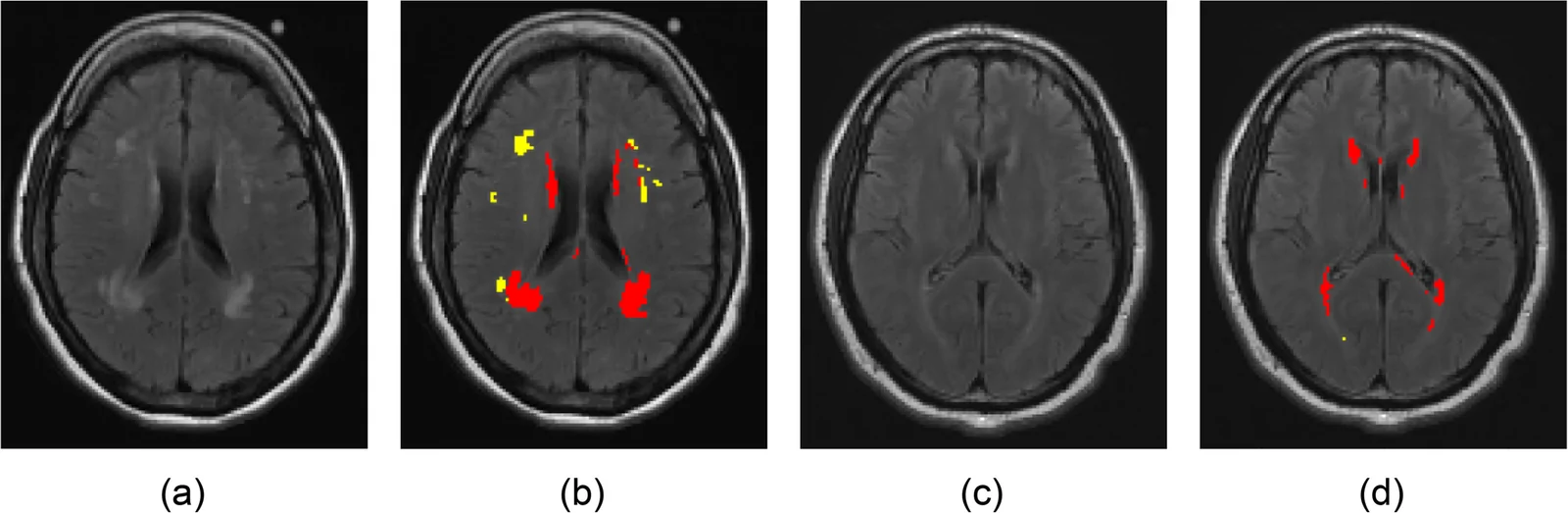
Axial FLAIR images and corresponding segmented WMHs using UBO detector from a representative HIV-only (**a**, **b**) and CU-only participant (**c**, **d**). pvWMHs and dWMHs are shown in red and yellow colors respectively

The quantification of WMH from the FLAIR images was carried out using the Unidentified Bright Objects (UBO) Detector with default parameter settings (Jiang et al. 2018). The UBO Detector is a cluster-based, fully automated machine-learning pipeline that has been found to be concordant with the gold standard methods of both manual tracing and the Fazekas visual rating scale for providing the location and severity of WMH (Hotz et al. 2022; Jiang et al. 2018). First, the FLAIR images were coregistered to the high-resolution T1-weighted images. Further, T1 images were warped to the existing DARTEL template for adults < 55 years old, and each individual T1 along with coregistered FLAIR were aligned to this template. This was followed by non-brain tissue removal and segmentation using FAST algorithm. In the UBO Detector pipeline, FAST is applied to FLAIR images using a three-class segmentation model (WMH, GM/WM, and CSF), exploiting the hyperintense appearance of WMH relative to surrounding brain tissue on FLAIR images. These candidate WMH clusters were subsequently classified using k-nearest neighbor clustering, a supervised machine-learning algorithm, to generate WMH probability maps. (Jiang et al. 2018). In this study, the default k-value of 5 and the built-in training set were used. A probability threshold of 0.7 was then applied to the probability maps to generate WMH segmentation masks. Further, a default distance threshold of 12 mm from the ventricular borders was employed to produce optimal segmentation of pvWMH and dWMH. All WMH segmentation masks underwent visual quality-control inspection to evaluate image registration and segmentation accuracy prior to further analyses. The total volume in mm^3^ was then computed for the pvWMH, dWMH, and total WMH burden. Figure 1 shows the raw FLAIR images and the UBO-generated maps of pvWMH and dWMH for a representative participant in HIV-only and MJ-only groups.

Volumetric segmentation of the acquired T1-weighted images was performed using FreeSurfer version 7.3.2 to obtain the estimated total intracranial volume (Fischl 2012). The segmented WMH volumes were divided by the total intracranial volume to obtain WMH load, which were then transformed for further statistical analysis.

### Statistical analysis

All statistical tests were conducted in SPSS 29. Descriptive statistics were used to characterize the sample on demographic and clinical characteristics. To test group differences, we used one-way ANOVA or independent sample t-test, as appropriate, for continuous variables and chi-squared tests for categorical variables. For non-normally distributed continuous variables, Kruskal-Wallis test or Mann-Whitney U Test was performed.

Age, sex, scanner software version, and CVD risk score were selected as covariates based on prior evidence demonstrating associations between these factors and WMH load. These variables were included to account for potential confounding when evaluating the effects of HIV and cannabis use. A linear regression model was used to evaluate the association of WMH load with age, sex, scanner software version, and CVD risk score. Although parameter estimates for these variables are reported for transparency, they were included as adjustment variables rather than primary predictors of interest. The independent and interactive effects of HIV and cannabis use on WMH load were evaluated using a 2 × 2 between-subjects ANCOVA model, controlling the associated covariates. Further, partial correlations were employed to examine the association of WMH load to global and domain-specific T scores, again controlling for age and scanner software version. A *p*-value of less than 0.05 was considered to be significant for all statistical tests.

## Results

### Sample characteristics

The sample included 296 participants across the four study groups (74 control, 73 CU-only, 76 HIV-only, and 73 HIV + CU). Participants were primarily African American (57%) and male (73%) with a mean age and education of 39.84 and 15.12 years, respectively. Table 1 summarizes the group comparisons on key clinical and demographic variables. The groups were comparable on sex and race. However, the mean age (*p* = 0.02) and years of education (*p* < 0.01) was significantly lower in people who use cannabis irrespective of HIV status. The CVD risk score ranged from 0 to 4 with a mean score of 1.37 (SD = 1.16), and there were no differences across the groups. Among the CVD risk factors, obesity (41%), hypertension (33%), nicotine usage (29%), and hyperlipidemia (28%) were found to be the most prevalent. Participants in CU groups were more likely to use nicotine (*p* < 0.001). Only 7% of the total participants had diabetes. On average, participants with HIV were diagnosed for a mean of 12 years and had been on ARV medications for 10 years, with no difference by cannabis status. The two HIV groups had similar duration of infection and ARV medication, as well as nadir and current CD4^+^ T-cell count. Only 3 of 152 (2.0%) participants had plasma HIV RNA levels in the range of 50–199 copies/mL, with all cases occurring in the HIV-only group.

**Table 1:** Demographic and clinical characteristics of participants

	Control (*N* = 74)	CU only (*N* = 73)	HIV only (*N* = 76)	HIV + CU (*N* = 73)	Statistic
Demographic and health characteristics:					
Age in years, M (SD)	40.50 (8.37)	37.64 (7.76)	41.42 (8.14)	39.82 (8.89)	F (3,292) = 2.78*
Male sex, n (%)	54 (73.00)	54 (74.00)	51 (67.10)	60 (82.20)	*Χ*^*2*^ (3) = 4.46
Race, n (%)					*Χ*^*2*^ (6) = 3.04
African American	44 (59.50)	44 (60.30)	41 (53.90)	40 (54.80)	
White	25 (33.80)	20 (27.40)	25 (32.90)	26 (35.60)	
Other mixed	5 (6.80)	9 (12.30)	10 (13.20)	7 (9.60)	
Education in years, M (SD)	16.00 (2.17)	14.68 (2.39)	15.41 (2.47)	14.41 (2.39)	F (3,292) = 6.83*
Days of alcohol use in past 30, M (SD)	4.26 (6.93)	6.03 (7.84)	4.16 (6.63)	6.29 (7.63)	F (3,292) = 1.80
Neuropsychological assessments:					
Executive function	53.44 (6.68)	50.48 (6.19)	50.88 (6.35)	50.89 (5.21)	F (3,292) = 3.60*
Learning	50.18 (8.45)	46.96 (9.12)	45.98 (10.70)	43.69 (8.49)	F (3,292) = 6.27*
Memory	51.83 (8.05)	48.51 (9.54)	48.33 (10.37)	45.21 (9.42)	F (3,292) = 6.13*
Working memory	52.94 (6.95)	52.52 (7.78)	49.32 (8.62)	49.95 (7.89)	F (3,292) = 3.99*
Global T-score	52.10 (6.21)	49.62 (6.71)	48.64 (7.81)	47.43 (6.50)	F (3,292) = 6.177*
Vascular risk factors:					
Diabetes, n (%)	8 (10.81)	4 (5.47)	6 (7.89)	2 (2.74)	*Χ*^*2*^ (3) = 4.14
Hypertension, n (%)	28 (37.83)	21 (28.76)	26 (34.21)	22 (30.14)	*Χ*^*2*^ (3) = 1.69
Hyperlipidemia, n (%)	25 (33.78)	14 (19.17)	27 (35.53)	16 (21.91)	*Χ*^*2*^ (3) = 7.55
Obesity, n (%)	26 (35.13)	30 (41.09)	36 (47.36)	29 (39.72)	*Χ*^*2*^ (3) = 2.37
Any nicotine use in past 30 days, n (%)	6 (8.10)	35 (47.94)	6 (7.89)	39 (53.42)	*Χ*^*2*^ (3) = 65.93*
CVD risk score, M (SD)	1.26 (1.28)	1.42 (1.14)	1.33 (1.02)	1.48 (1.18)	H (3) = 2.23
HIV characteristics:					
Years since diagnosis, M (SD)	-	-	11.73 (7.16)	12.61 (7.93)	t (147) = -0.71
Current CD4 > 500/mL, n (%)	-	-	63 (82.90)	66 (90.40)	*Χ*^*2*^ (1) = 1.81
Nadir CD4 count, Mdn (IQR)	-	-	284.50 (257.75)	347.50 (390.25)	U = 2870.00
Years since ARV initiation, M (SD)	-	-	10.30 (6.41)	10.56 (7.59)	t (147) = -0.22
Cannabis use characteristics:					
Years of regular use, M (SD)	-	12.93 (8.17)	-	13.47 (9.01)	t (144) = -0.25
Age of first use, M (SD)	-	22.40 (7.28)	-	23.42 (7.28)	t (143) = -0.84
Days of use in past 30, M (SD)	-	24.47 (7.20)	-	25.67 (7.38)	t (144) = -0.76
Route of administration, n (%)					*Χ*^*2*^ (2) = 0.36
Smoking	-	52 (71.20)	-	53 (73.60)	
Oral	-	2 (2.70)	-	1 (1.40)	
Both	-	19 (26.00)	-	18 (25.00)	
Hours high per day, M (SD)	-	5.29 (3.81)	-	4.99 (4.01)	t (143) = 0.77

CU – Cannabis use, SD – Standard deviation, IQR – Interquartile range* *p* < 0.05

Participants in the CU groups had 13.2 years of regular use on average and began using at a mean age of 23 years. In the past 30 days, they used cannabis on 24.92 days and were high for an average of 5.10 h per day. Smoking was reported as the primary route of administration (72%), while 25% of participants reported both smoking and oral administration. There were no differences in these cannabis use characteristics by HIV status. Measures of recent alcohol use (days drinking alcohol in the past 30 days) did not differ significantly across the four study groups (*p* > 0.05).

The distribution of scanner software versions was comparable across groups, with Version 27 accounting for 62.2% (46/74) of controls, 71.2% (52/73) of the CU-only group, 60.5% (46/76) of the HIV-only group, and 68.5% (50/73) of the HIV + CU group. A chi-square test indicated no significant association between group status and scanner software version, χ²(3) = 2.55, *p* = 0.466.

### WMH load by HIV status and cannabis use

The overall burden of WMH was fairly low in the sample, with the raw volumes ranging from 101.25 to 27,698.63 mm^3^ (M = 2,571.75, SD = 2,645.97), equivalent to a WMH load of 0.01% to 1.80% of estimated total intracranial volume, respectively. The majority of the WMH burden was along the periventricular region rather than deep white matter, with mean volumes of 1827.39 and 703.45 mm^3^, respectively. Consistent with their selection as covariates, age and sex were significantly associated with both periventricular and deep WMH load, with greater burden observed at older ages and among females (Table 2). The WMH load was also influenced by the software version of the scanner. Furthermore, CVD risk score was associated with increased pvWMH load, but not dWMH load. Consequently, these predictor variables of WMH load were accounted for further analysis. Although association of these variables with WMH load are reported for transparency, they were included as adjustment variables rather than primary predictors of interest.

**Table 2:** Results of linear regression model for identifying predictors of WMH load

	pvWMH load		dWMH load	
	Standardized coefficient (β)	*p*-value	Standardized coefficient (β)	*p*-value
Age	0.294	< 0.001*	0.331	< 0.001*
Male sex	-0.165	0.001*	-0.165	< 0.001*
Scanner software version	0.226	< 0.001*	0.418	< 0.001*
CVD risk score	0.217	< 0.001*	0.063	0.210

**Table 3:** Independent and interactive effects of HIV and cannabis use on WMH load

	Main effect of HIV		Main effect of CU		HIV×CU effect	
	F-statistic	*p*-value	F-statistic	*p*-value	F-statistic	*p*-value
pvWMH load	0.266	0.61	2.644	0.10	8.703	0.003*
dWMH load	0.288	0.59	0.290	0.59	0.0002	0.99

**Fig. 2:**
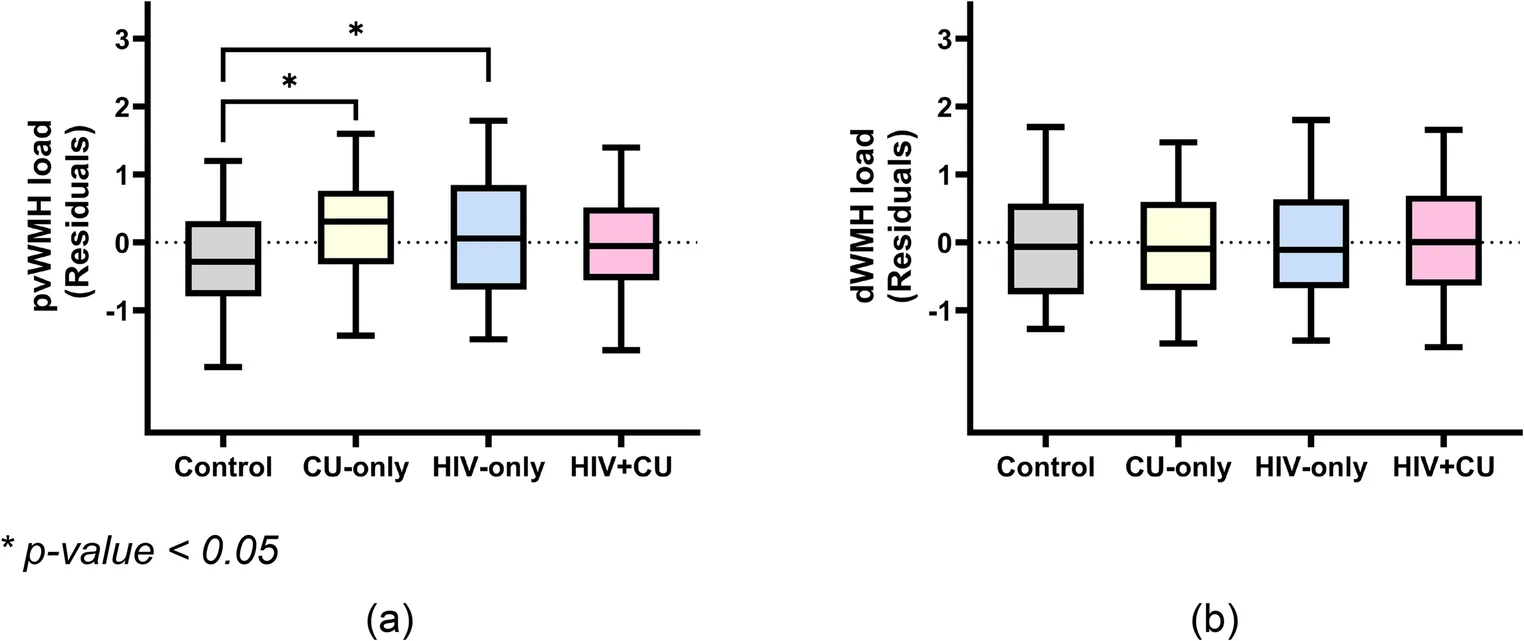
Adjusted (**a**) pvWMH and (**b**) dWMH load of controls, CU-only, HIV-only and HIV + CU groups. Solid line within each box represents the median value, with whiskers extending to the 5th and 95th percentiles of data distribution

The results of the 2 × 2 between-subjects ANCOVA models are summarized in Table 3. There were no main effects of HIV or cannabis for both pvWMH and dWMH load. However, a significant interaction effect of HIV and cannabis use was found for pvWMH load. Complete ANCOVA results, including covariate effects and effect size estimates (partial η²), are provided in Supplementary Tables S1-S2. The overall model explained 29.6% of the variance in pvWMH load (R² = 0.296; adjusted R² = 0.279) and 34.9% of the variance in dWMH load (R² = 0.349; adjusted R² = 0.333). For pvWMH load, the HIV × MJ interaction demonstrated a small-to-moderate effect size (partial η² = 0.029), whereas the main effects of HIV (partial η² = 0.001) and CU (partial η² = 0.009) were negligible. For dWMH load, effect sizes for HIV, CU, and their interaction were minimal (partial η² ≤ 0.001). Figure 2 shows the box plot representation of WMH load after adjusting for the identified covariates. Post-hoc analysis revealed that the pvWMH loads in the CU and HIV-only groups were significantly higher than the controls as seen in Fig. 2 (a). In contrast, no difference was found between these volumes of HIV + CU and controls. Similarly, as observed from Fig. 2 (b), there were no significant differences among the groups for dWMH load.

### Association between pvWMH load and cognitive functions

**Fig. 3:**
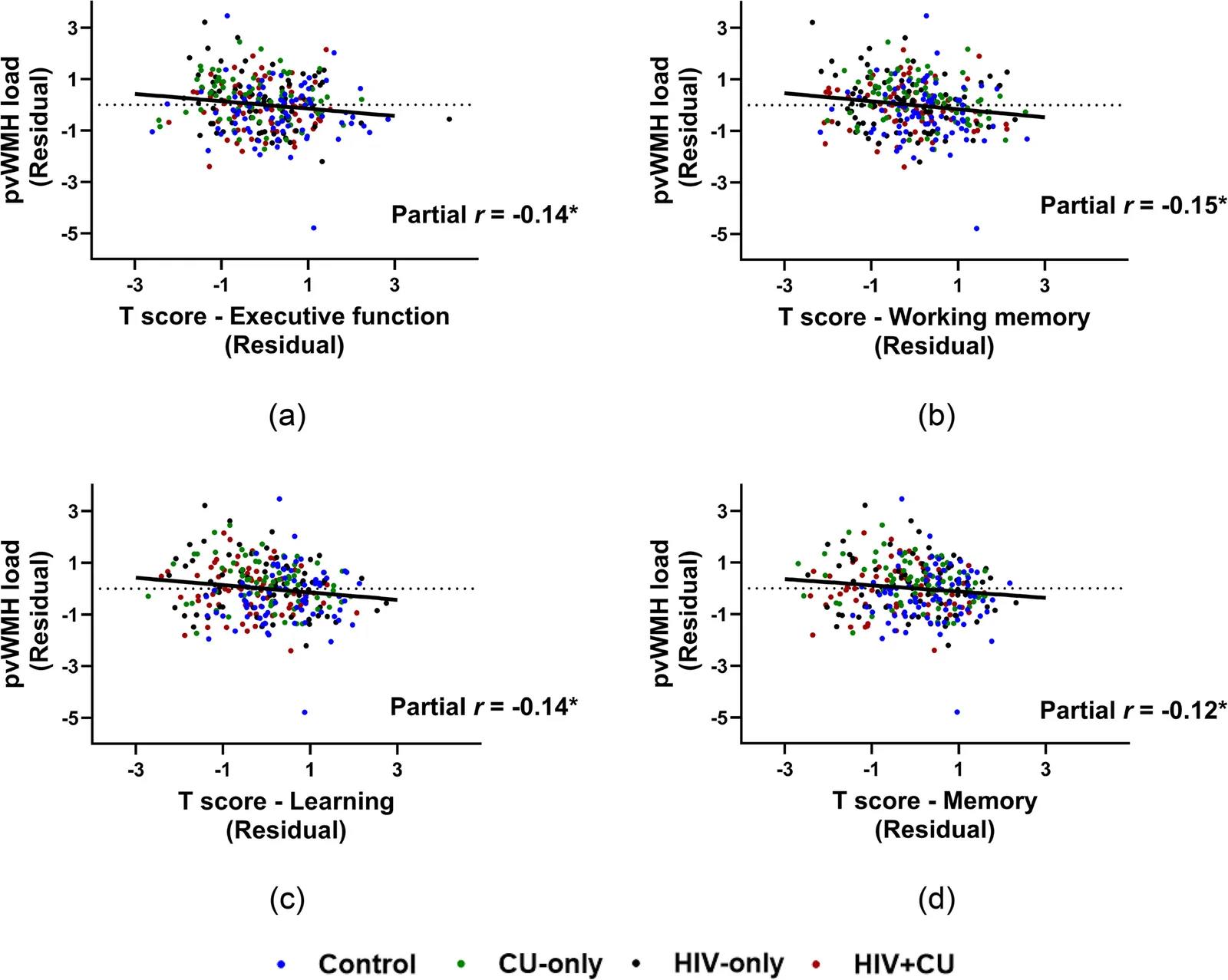
Scatter plot of partial correlation between pvWMH load and T scores of (**a**) executive function (**b**) working memory, (**c**) learning and (**d**) memory for all subjects

Partial correlation analyses showed that pvWMH load was negatively correlated with global T score for all the subjects (r_partial_ = -0.16, *p* = 0.004). These associations were maintained for all domain-specific T scores: pvWMH load was significantly associated with executive function (r_partial_ = -0.14, *p* = 0.01), working memory (r_partial_ = -0.15, *p* = 0.007), learning (r_partial_ = -0.14, *p* = 0.01) and memory (r_partial_ = -0.12, *p* = 0.04), as seen in Fig. 3.

## Discussion

This study examined the effect of cannabis use on WMH load in treated PLWH who had sustained viral suppression. Our principal findings demonstrate an interaction effect of HIV and cannabis use on the pvWMH load, but not dWMH. Specifically, pvWMH loads were higher in HIV-only and CU-only groups compared to controls, supporting independent effects. Interestingly, these WMHs were not elevated in persons with co-occurring HIV and cannabis use relative to controls, driving the observed interaction effect. This effect persisted after accounting for age, sex, and CVD risk factors. The similarity in pvWMH load for the HIV + CU group and controls implies the potential mitigating effect of cannabis on neuroinflammation caused by HIV infection (Ellison et al. 2026; Langat et al. 2025; Manuzak et al. 2018). In our examination of cognitive test scores, higher pvWMH load was associated with poorer cognitive function, both globally and in the domains of executive function, memory, learning, and working memory.

The overall burden of pvWMH was higher than dWMH in our sample, an observation that is consistent with the existing literature (Chung et al. 2025; Griffanti et al. 2018). This may reflect the vulnerability of periventricular white matter to vascular injury. These regions are supplied by penetrating arteries with limited collateral circulation and are located within watershed zones between major arterial territories, making them particularly susceptible to ischemia and chronic hypoperfusion (Duering et al. 2023; Ye et al. 2025). Consequently, the predominance of WMHs in periventricular regions may be attributable to their increased vulnerability to cerebrovascular injury, in addition to other mechanisms related to ventricular proximity and white matter microstructural alterations. Furthermore, the observed effects of HIV and cannabis were confined to pvWMH and absent in dWMH, indicating localized alterations near the ventricles rather than diffusely throughout the white matter tracts. One possible explanation is that periventricular regions may be particularly vulnerable to processes associated with vascular injury and white matter degeneration. Histopathologically, pvWMH is characterized by gliosis, loosening of the white matter fibers, and myelin loss, whereas dWMH is heterogenous and could represent axonal loss and vacuolation (Griffanti et al. 2018; Wharton et al. 2015). However, our findings do not establish that pvWMH are more specifically associated with HIV-related neuroinflammation than dWMH. As periventricular lesions often emerge earlier and are more prevalent than deep white matter lesions in middle-aged adults, the lack of significant dWMH findings may reflect a lower burden and reduced variability of deep white matter lesions in this cohort, limiting our ability to detect associations in those regions. Future studies including participants with a wider range of white matter disease burden and longitudinal follow-up are needed to clarify whether HIV- and cannabis-related effects differentially influence periventricular and deep WMH development.

Our findings indicate that pvWMH load was significantly increased in HIV-only group compared to controls. These are concordant with existing studies (McMurtray et al. 2008; Mina et al. 2021; Su et al. 2016). The higher WMH accumulation in PLWH could be related to persistence of viral reservoirs in the central nervous system, having an impact on the brain parenchyma, contributing to chronic neuroinflammation and increased cerebrovascular damages (Ellis et al. 2023; Jia and Brew 2025; Mina et al. 2021). We did not find a significant link between WMH load and HIV disease characteristics, such as recent and nadir CD4 counts, but the majority of participants had immune reconstitution with CD4 T-cell counts above 500. Our participants were also presented with relatively homogeneous HIV disease profile, as all were on combination ART and had sustained HIV viral suppression. These findings align with the recent studies suggesting that, in the era of ART, the direct influence of viral load on the brain integrity may be diminished (Moschopoulos et al. 2024; Trentalange et al. 2020; Watson et al. 2017).

Our primary finding was a significant interaction between HIV status and cannabis use on pvWMH load. Specifically, among participants who do not use cannabis, PLWH exhibited significantly higher WMH burden than HIV-negative participants, whereas the corresponding HIV-related difference was attenuated and no longer statistically significant among participants who use cannabis. This pattern is consistent with the possibility that cannabis use modifies the relationship between HIV infection and white matter abnormalities. However, because direct comparisons between PLWH who do and do not use cannabis were not statistically significant, the current findings do not provide evidence that cannabis use directly reduces WMH burden among PLWH. (Manuzak et al. 2018)., Nevertheless, the current literature lacks consensus on the protective effects of cannabis in the context of HIV (Chen et al. 2026). Cannabinoids present in cannabis are reported to modulate neuroinflammation by suppressing the production of pro-inflammatory cytokines while simultaneously promoting anti-inflammatory ones, thereby influencing the immune cell activity (Ellison et al. 2026; Langat et al. 2025; Watson et al. 2021). Cannabinoids also possess antioxidant properties that mitigate neuroinflammation and promote neurogenesis (Marsicano et al. 2002). They also demonstrate the ability to reduce reactive gliosis and neuronal damage. Studies detailing the increased expression of cannabinoid receptors in HIV-associated neurocognitive disorders, indicate that cannabinoid-based interventions could be a promising therapeutic pathway for HIV (Manuzak et al. 2018). Further investigation into the specific mechanism underlying this potential protective effect is warranted.

Importantly, the observed interaction should not be interpreted as evidence that cannabis is uniformly neuroprotective in the context of HIV. Rather, our findings suggest that the relationship between cannabis use and WMH burden may differ according to HIV status, potentially reflecting complex interactions between HIV-associated neuroinflammation and cannabinoid signaling pathways. Additionally, cannabis is a heterogeneous substance, and its biological effects may vary according to strain, potency, and cannabinoid composition (Senator et al. 2025). Because data on cannabis strain, THC/CBD content, and product potency were not available in the present study, we were unable to evaluate their potential contribution to the observed findings. Future studies incorporating detailed cannabinoid exposure measures are needed to better understand these relationships.

Conversely, in participants without HIV, cannabis use was associated with higher pvWMH load compared to controls. This finding is consistent with emerging literature reporting reduced white matter integrity in people who use cannabis, potentially reflecting the myelin loss and cerebrovascular damage (Cousijn et al. 2022; Orr et al. 2016). These results imply that cannabis may not consistently offer protection against WMH. Emerging studies suggest that contributing factors may include high-potency tetrahydrocannabinol present in cannabis, age of regular usage onset, and the cumulative effect of use duration and frequency over time (Manuzak et al. 2018; Ricci et al. 2026).

To understand the clinical relevance of WMH, we analyzed their correlation with cognitive measures. Consistent with the existing literature, increased pvWMH load was associated with impaired cognitive functions (Chien et al. 2024; Griffanti et al. 2018; Thames et al. 2017; Wang et al. 2022). In particular, the pvWMH was associated with executive, memory, learning, and working memory domains. This is in line with the notion that these higher order functions rely on long distance connections in the white matter, which are more likely to be disrupted by pvWMHs (Griffanti et al. 2018). The association between WMH load and cognitive performance was examined across the full sample to maximize power and variability in the outcome measures. Future studies with larger samples should investigate whether the relationship between WMH and cognition differs as a function of HIV status and cannabis use, as such effects may provide additional insight into the neurobiological significance of WMH accumulation in these populations.

Age, sex, and CVD risk factors were included as covariates because prior studies have consistently identified these factors as important contributors to WMH load (Gupta et al. 2018; Holroyd et al. 2025; Mina et al. 2021). Within our adjusted models, older age and female sex were significantly associated with increases in both periventricular and deep WMH. It is notable that there was a considerable burden of pvWMH, despite the relatively young age of the sample. However, because the study was not designed or powered to evaluate demographic differences, including sex, as a primary research question, the observed associations with WMH load should be interpreted cautiously. There was also positive association between CVD risk score and pvWMH load. This aligns with the understanding that CVD risk factors such as hypertension, diabetes, hyperlipidemia and nicotine usage can lead to small vessel damage in the brain which eventually manifests as WMHs (Mina et al. 2021). Interestingly, this association was absent for dWMH. Although this pattern is consistent with prior reports suggesting that vascular risk factors may have a stronger relationship with periventricular than deep white matter abnormalities, (Gronewold et al., 2022) the present study was not designed to formally test these associations. Since cardiovascular risk factors are linked to both HIV, cannabis use and WMH load, adjusting for CVD risk helps determine whether HIV and cannabis use are associated with WMH load independently of traditional vascular risk factors.

This study is among the first to investigate the effect of cannabis on WMH load at specific brain locations in virally suppressed individuals with HIV. Additional strengths include comprehensive assessments to characterize the sample and rigorous eligibility criteria related to cannabis use and HIV disease. However, this study also has limitations. Although we employed UBO detector, which is a standardized and validated tool for WMH quantification, it is inherently limited by accuracy and reliability. While we strive to maintain rigor in the application of this method by performing manual quality checks for every individual WMH segmentation map, it remains possible that results might vary across methods. Similarly, while our automated WMH detection technique allowed for detailed quantification of WMH volumes using voxels, many of these changes may not be significant enough to be noted on visual inspection of FLAIR sequences. Further, although WMH burden in our sample may be broadly contextualized using large cohorts, direct comparisons of absolute WMH volumes should be interpreted cautiously due to substantial differences in participant age, MRI acquisition protocols, and image processing methods across studies. The generalizability of our findings may be limited by our relatively young, well-educated sample compared with the broader population of PLWH in the United States. Additionally, although the neuropsychological battery assessed several key cognitive domains, the present analysis did not evaluate processing speed, motor function, or social cognition. Thus, the implications of this work for clinical outcome measures require further investigation. While we identified a strong association between CVD risk and WMH load, it should be noted that participants with a history of cerebrovascular events such as stroke were excluded in this study. Finally, as this is a cross-sectional study, longitudinal designs may be needed to establish the degree to which HIV and cannabis use are causal factors in the development and progression of WMHs. Future studies should also examine exposure to specific cannabinoids, including THC and CBD, and levels of metabolites in circulating blood.

In conclusion, our study confirms that HIV disease and cannabis use can each increase pvWMH load, but that cannabis use among PLWH may modulate effects. This interaction effect persisted even after accounting for cardiovascular risk factors and age. Our findings also demonstrate higher WMH burden to be associated with poorer cognitive performance, underscoring its relevance to HIV-associated neurocognitive disorders. However, given that cannabis use is associated with harms, including worsened cognitive function, it is critical that this research be replicated before making definitive clinical recommendations. Thus, this study establishes a critical foundation for future studies examining effects of cannabis use characteristics on white matter injury and underlying mechanisms in people living with HIV.

## Supplementary Information

Below is the link to the electronic supplementary material.

Supplementary Material 1

## Data Availability

The data that supports the findings of this study are available from the corresponding author upon reasonable request.
