# Supplementary material for: Impact of cannabis use on white matter hyperintensities in adults with and without chronic HIV disease
Source: J Neurovirol. 2026 Aug 27;32(5):38. doi: 10.1007/s13365-026-01338-2 (PMC13522075; doi:10.1007/s13365-026-01338-2)
Supplement: Supplementary file 1 [file 13365_2026_1338_MOESM1_ESM.docx]

Supplementary Table S1. Full ANCOVA model for periventricular white matter hyperintensity (pvWMH) load, including covariates, effect sizes (partial η²), and model fit statistics.

|  | Type III Sum of Squares | df | Mean Square | F | Sig | Partial Eta squared |
| --- | --- | --- | --- | --- | --- | --- |
| Corrected model | 9.247 | 7 | 1.321 | 17.322 | < 0.001 | 0.296 |
| Intercept | 5.449 | 1 | 5.449 | 71.449 | < 0.001 | 0.199 |
| Age | 2.574 | 1 | 2.574 | 33.749 | < 0.001 | 0.105 |
| Sex | 0.755 | 1 | 0.755 | 9.899 | 0.002 | 0.033 |
| Scanner software version | 1.682 | 1 | 1.682 | 22.050 | < 0.001 | 0.071 |
| CVD risk score | 1.150 | 1 | 1.150 | 15.083 | < 0.001 | 0.050 |
| HIV | 0.020 | 1 | 0.020 | 0.266 | 0.607 | 0.001 |
| MJ | 0.202 | 1 | 0.202 | 2.644 | 0.105 | 0.009 |
| HIV × MJ | 0.664 | 1 | 0.664 | 8.703 | 0.003 | 0.029 |
| Error | 21.963 | 288 | 0.076 |  |  |  |
| Total | 2780.252 | 296 |  |  |  |  |
| Corrected total | 31.210 | 295 |  |  |  |  |

R squared = 0.296 (Adjusted R squared = 0.279)

Supplementary Table S2. Full ANCOVA model for deep white matter hyperintensity (dWMH) load, including covariates, effect sizes (partial η²), and model fit statistics.

|  | Type III Sum of Squares | df | Mean Square | F | Sig | Partial Eta squared |
| --- | --- | --- | --- | --- | --- | --- |
| Corrected model | 0.011 | 7 | 0.002 | 22.085 | < 0.001 | 0.349 |
| Intercept | 0.005 | 1 | 0.005 | 71.741 | < 0.001 | 0.199 |
| Age | 0.003 | 1 | 0.003 | 39.333 | < 0.001 | 0.120 |
| Sex | 0.001 | 1 | 0.001 | 11.446 | < 0.001 | 0.038 |
| Scanner software version | 0.005 | 1 | 0.005 | 75.545 | < 0.001 | 0.208 |
| CVD risk score | 0.000 | 1 | 0.000 | 1.703 | 0.193 | 0.006 |
| HIV | 2.037E-5 | 1 | 2.037E-5 | 0.288 | 0.592 | 0.001 |
| MJ | 2.046E-5 | 1 | 2.046E-5 | 0.290 | 0.591 | 0.001 |
| HIV × MJ | 1.399E-8 | 1 | 1.399E-8 | 0.000 | 0.989 | 0.000 |
| Error | 0.020 | 288 | 7.064E-5 |  |  |  |
| Total | 0.138 | 296 |  |  |  |  |
| Corrected total | 0.031 | 295 |  |  |  |  |

R squared = 0.349 (Adjusted R squared = 0.333)
